# Effects of pre-existing type 1 diabetes mellitus on survival outcome following out-of-hospital cardiac arrest: a registry-based observational study in Sweden

**DOI:** 10.1136/bmjopen-2023-080710

**Published:** 2024-07-15

**Authors:** Berkan Eken, Araz Rawshani, Aidin Rawshani, Zacharias Mandalenakis, Erik Thunstrom, Antros Louca, Petur Petursson, Oskar Angerås, Sadek Nadhir, Christian Dworeck, Truls Råmunddal

**Affiliations:** 1 Sahlgrenska Academy, University of Gothenburg, Gothenburg, Sweden; 2 Department of Molecular and Clinical Medicine, Institute of Medicine, Gothenburg, Sweden

**Keywords:** Cardiopulmonary Resuscitation, General diabetes, Out-of-Hospital Cardiac Arrest

## Abstract

**Abstract:**

**Background:**

It has been estimated that 80% of cases of out-of-hospital cardiac arrest (OHCA) are due to cardiac causes. It is well-documented that diabetes is a risk factor for conditions associated with sudden cardiac arrest. Type 1 diabetes (T1D) displays a threefold to fivefold increased risk of cardiovascular disease and death compared with the general population.

**Objective:**

This study aims to assess the characteristics and survival outcomes of individuals with and without T1D who experienced an OHCA. **Design**: A registry-based nationwide observational study with two cohorts, patients with T1D and patients without T1D. **Setting**: All emergency medical services and hospitals in Sweden were included in the study.

**Participants:**

Using the Swedish Cardiopulmonary Resuscitation Registry, we enrolled 54 568 cases of OHCA where cardiopulmonary resuscitation was attempted between 2010 and 2020. Among them, 448 patients with T1D were identified using International Classification of Diseases-code: E10.

**Methods:**

Survival analysis was performed using Kaplan-Meier and logistic regression. Multiple regression was adjusted for age, sex, cause of arrest, prevalence of T1D and time to cardiopulmonary resuscitation.

**Main outcome measures:**

The outcomes were discharge status (alive vs dead), 30 days survival and neurological outcome at discharge.

**Results:**

There were no significant differences in patients discharged alive with T1D 37.3% versus, 46% among cases without T1D. There was also no difference in neurological outcome. Kaplan-Meier curves yielded no significant difference in long-term survival. Multiple regression showed no significant association with survival after accounting for covariates, OR 0.99 (95% CI 0.96 to 1.02), p value=0.7. Baseline characteristics indicate that patients with T1D were 5 years younger at OHCA occurrence and had proportionally fewer cases of heart disease as the cause of arrest (57.6% vs 62.7%).

**Conclusion:**

We conclude, with the current sample size, that there is no statistically significant difference in long-term or short-term survival between patients with and without T1D following OHCA.

**Strengths and limitations of this study**

- The study sample is representative of out-of-hospital cardiac arrest cases where resuscitation is attempted since >90% are recorded in the registry.
- The sample includes all regions in Sweden and the risk of selection bias is minimal.
- The sample size is relatively small, with 448 patients with type 1 diabetes.
- We did not have access to greater details regarding diabetes diagnosis; diagnoses were based on International Classification of Diseases codes with the risk of misclassification and coding errors.
- The Swedish Cardiopulmonary Resuscitation Registry does not include patients for whom resuscitation is deemed futile, excluding a cohort that may contain patients with type 1 diabetes.

## Introduction

 Out-of-hospital cardiac arrest (OHCA) is a leading cause of mortality worldwide.^1^ The European Registry of Cardiac Arrest identifies OHCA as the third leading cause of death in Europe.^2^ It has been estimated that 80% of cases of OHCA are due to cardiac causes, primarily coronary artery disease in its various forms.^3^ OHCA in the younger population typically has different aetiologies, trauma, intoxications and suicide attempts being much more common than in older adults and the elderly. Cardiovascular diseases, including cardiomyopathies, channelopathies, myocarditis and coronary artery anomalies, do occur but are not common at a population level.^4^

It is well documented that diabetes is a risk factor for virtually all conditions associated with sudden cardiac arrest (SCA), including coronary artery disease, myocardial infarction and heart failure. Previous studies have reported a twofold to fourfold increase in the risk of SCA in patients with diabetes.^5^ Type 1 diabetes (T1D) exhibits a threefold to fivefold increased risk of cardiovascular disease (CVD) and death compared with the general population. Indeed, the risk for CVD and death in T1D is doubled even with glycated haemoglobin levels at or below target levels.^6^

While there are large studies exploring the association between type 2 diabetes (T2D) and OHCA outcomes, we are unaware of a study as large as the current one for T1D. Often conjoined in similar studies, T2D and T1D are, however, significantly different. T1D is generally characterised by insulin deficiency emanating from the immune-mediated destruction of insulin-producing pancreatic beta cells.^7^ In contrast, T2D is a progressive metabolic disease marked by insulin resistance and eventual failure of pancreatic beta cells.^8^ Although the outcome for both conditions is characterised by hyperglycaemia, each type is often burdened with different comorbidities requiring distinct treatment approaches.

The aim of the study was to assess the characteristics and outcomes of individuals with T1D who experienced an OHCA, compared with patients without T1D experiencing an OHCA.

## Methods

We conducted a nationwide observational study including all cases of OHCA recorded between 2010 and 2020 in the Swedish Cardiopulmonary Resuscitation Registry (SCRR); in total 54 586 patients were recorded. The registry was initiated in 1990 and includes more than 90% of all OHCA cases where resuscitation is attempted. Cases where resuscitation is deemed futile are not included in the registry. In Sweden, emergency medical services (EMS) are provided both by the respective province (public) and by private companies. However, all ambulance organisations throughout the nation participate in the registry. Reporting is done prospectively and uses the Utstein-based template. The annual report is available at www.shlr.registercentrum.se.

We merged the SCRR with the patient registry, which includes both inpatient and outpatient reports throughout Sweden. The patient registry is managed by the Swedish National Board of Health and Welfare, with coverage since 1987, and currently achieves a 100% level of ascertainment. All diagnoses since year 2000 were assessed, allowing for a 10-year period to record diabetes and other diagnoses. In Sweden, individuals with T1D undergo annual examinations, such that a 10-year period should allow for an adequate level of ascertainment with regard to diabetes status. Merging the SCRR with the patient registry is a seamless process due to the Swedish personal identity number, a unique 12-digit ID assigned to all citizens.

The patient registry uses the International Classification of Diseases (ICD) 10 codes. Diagnosis code E10 defines T1D, and E11 defines T2D. The absence of any of these codes defined a non-diabetic. The presence of only E10 defined a person with T1D, while the occurrence of only E11 or both E10 and E11 defined T2D. Only diagnoses established prior to the date of OHCA were assessed.

### Statistical analysis

Statistical calculations were performed using R Statistical Software (V.4.2.3; R Core Team 2023). Patient baseline characteristics are described using means and medians together, along with appropriate measurements of dispersion. Long-term survival comparisons between patients with and without T1D were made using unadjusted Kaplan-Meier estimates, followed by a log-rank test. Furthermore, adjusted logistic regression evaluated the binary outcome of 30 days survival. Adjusted models included the following covariates: age, sex, initial rhythm, cause of arrest and time from arrest to cardiopulmonary resuscitation (CPR) start. Descriptive models were used to demonstrate pre-arrest comorbidities between subpopulations.

The outcomes were discharge status (alive vs dead), 30-day survival and neurological outcome at discharge, which was classified using the cerebral performance category (CPC). The CPC score is a 5-point scale, where categories 1–2 are generally considered good neurological outcome and 3 or higher indicates a poor neurological outcome.

### Patient and public involvement statement

Patients or the public were not involved in the design, or conduct, or reporting, or dissemination plans of our research.

## Results

### Baseline characteristics

In total, 54 568 cases of OHCA were recorded. As shown in table 1, patients with T1D prior to OHCA accounted for 448 of the cases. Patients with T1D were approximately 5 years younger, with no differences in sex. Concerning causes of arrest, 57.6% of those with T1D had heart disease as the presumed cause versus 62.7% among patients without T1D. No major differences were found with respect to the location of arrest, with most cases occurring at home (72.6% in T1D vs 71.6%). Being born in Sweden was more common in people with T1D (89.7% vs 85.6%). There were differences regarding coexisting conditions prior to OHCA, such that people with T1D had significantly more hypertension (61.2% vs 44.7%), dyslipidaemia (28.6% vs 15.6%) and renal failure (15.2% vs 9.9%).

**Table 1:** Baseline characteristics in 54 568 patients with out-of-hospital cardiac arrest stratified by pre-exististence of type 1 diabetes

	Type 1 diabetes	No type 1 diabetes	P value
n	448	54 120	
Group - n (%)			
Sex women	163 (36.4)	18 427 (34.1)	0.335
Patient characteristics - mean (SD)			
Age	64.39 (17.24)	69.23 (17.51)	<0.001
Cause of cardiac arrest - n (%)			<0.001
Heart disease	228 (57.6)	30 158 (62.7)	
Overdose or intoxication	6 (1.5)	1381 (2.9)	
Trauma or accident	12 (3.0)	1112 (2.3)	
Pulmonary disease	14 (3.5)	2704 (5.6)	
Suffocation	10 (2.5)	1266 (2.6)	
Suicide	5 (1.3)	1098 (2.3)	
Drowning	0 (0.0)	456 (0.9)	
Other	121 (30.6)	9889 (20.6)	
**Time of cardiac arrest - n (%**)			0.001
00:00 to 06:00	85 (22.9)	7387 (16.4)	
13:00 to 18:00	119 (32.1)	13 652 (30.3)	
19:00 to 23:00	68 (18.3)	8630 (19.1)	
07:00 to 12:00	99 (26.7)	15 443 (34.2)	
**Location of cardiac arrest - n (%**)			0.779
Home	324 (72.6)	38 574 (71.6)	
Public place	67 (15.0)	8764 (16.3)	
Other places	55 (12.3)	6553 (12.2)	
Prehospital interventions - n (%)			
Bystander CPR*	240 (55.6)	28 645 (55.0)	0.844
Intubation performed	131 (29.6)	15 035 (28.3)	0.563
Defibrillated, any	137 (31.3)	17 356 (33.4)	0.382
Defibrillated, number of attempts - mean (SD)	3.43 (3.54)	3.48 (3.16)	0.859
Epinephrine administered	371 (83.6)	42 146 (78.8)	0.018
Amiodarone administered	42 (9.5)	6193 (11.8)	0.173
**Critical time intervals - median (IQR**)			
Time from arrest to EMS† dispatch	2.00 (1.00–5.00)	2.00 (1.00–5.00)	0.971
Time from arrest to CPR start	2.00 (0.00–10.00)	3.00 (0.00–10.00)	0.100
Time from arrest to defibrillation	17.00 (11.00–29.00)	15.00 (8.00–24.00)	0.005
Time from arrest to EMS arrival	13.00 (9.00–19.00)	13.00 (8.00–20.00)	0.900
Time from EMS dispatch to arrival	10.00 (7.00–16.00)	10.00 (7.00–16.00)	0.737
Time from arrest to ROSC‡	15.50 (10.00–23.75)	15.00 (9.00–23.00)	0.392
**Initial presentation - n (%**)			
**Initial rhythm**			0.357
Ventricular fibrillation/pulseless ventricular tachycardia	83 (20.9)	11 083 (23.2)	
Pulseless electrical activity	64 (16.1)	8224 (17.2)	
Asystole	251 (63.1)	28 439 (59.6)	
Consciousness on EMS arrival at scene	49 (11.1)	5588 (10.6)	0.803
Breathing on EMS arrival at scene			0.604
No breathing	339 (77.2)	40 852 (77.7)	
Agonal breathing	42 (9.6)	5651 (10.7)	
Normal breathing	58 (13.2)	6053 (11.5)	
Unknown	0 (0.0)	22 (0.0)	
Pulse on EMS arrival at scene	64 (15.0)	7200 (14.0)	0.614
Spontaneous circulation on hospital arrival	112 (46.1)	13 823 (44.9)	0.749
Consciousness on hospital arrival	23 (9.6)	3319 (11.0)	0.551
Witnessed cardiac	259 (59.4)	34 119 (65.0)	0.018

* Cardiopulmonary resuscitation.

† Emergency medical services.

‡ Return of spontaneous circulation.

EMSemergency medical service

Patients with T1D were more frequently prescribed ACE inhibitors and angiotensin receptor blockers (44.9% vs 32.7%), lipid-lowering drugs (36.8% vs 23.7%), calcium channel blockers (24.6% vs 15.9%) and diuretics (32.1% vs 27.0%) (online supplemental table 1).

Both groups received similar rates of bystander CPR (55.6% vs 55.0%). Patients with T1D received epinephrine at higher rates (83.6% vs 78.8%). Critical time intervals and initial rhythm, as presented in table 1, showed no significant differences between the subgroups. Patients without T1D had a higher degree of witnessed arrest (65.0% vs 59.4%).

**Figure 1:**
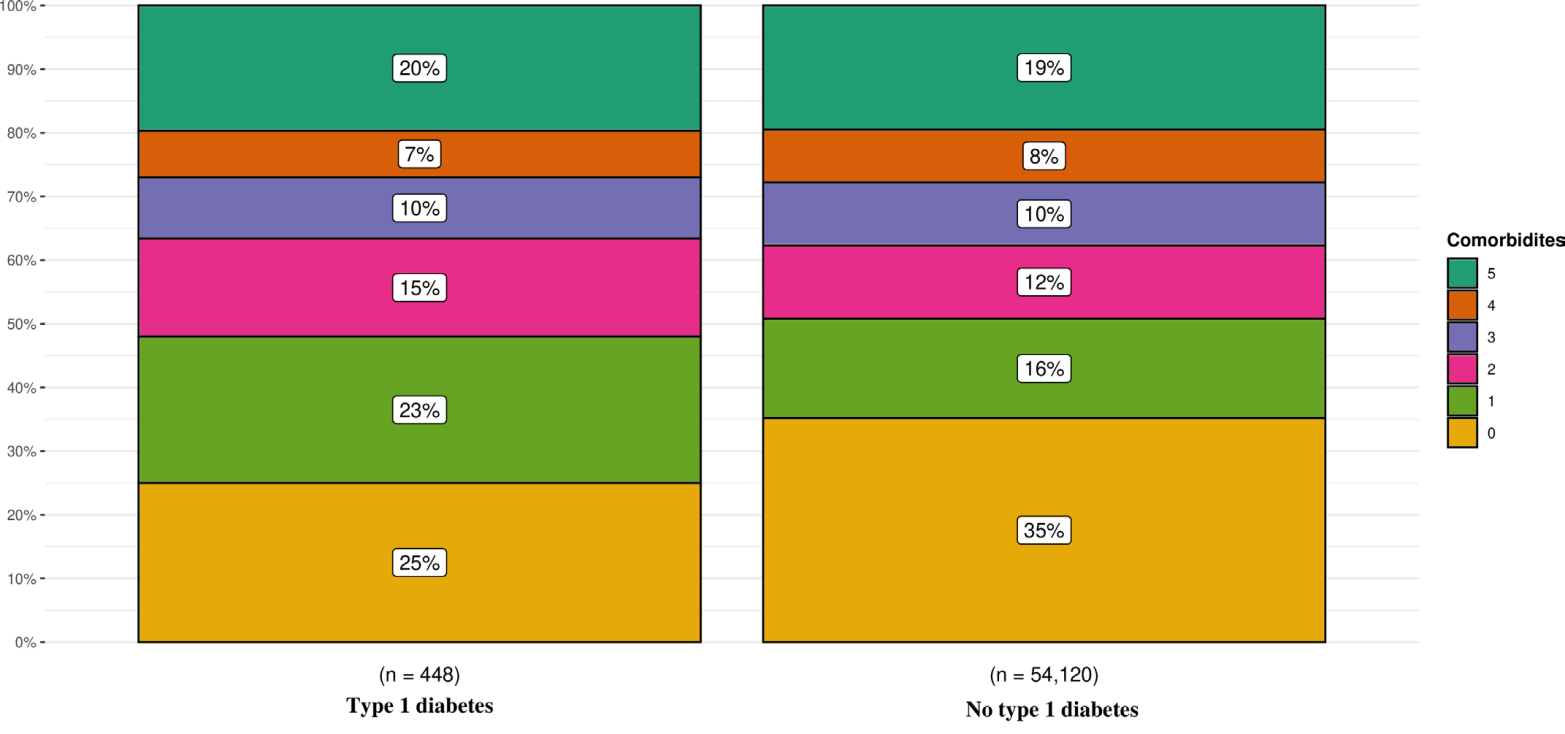
Proportion of the number of cardiovascular comorbidities.

Figure 1 displays the proportion of patients with cardiovascular comorbidities; 75% of patients with T1D had at least one or more cardiovascular comorbidity, and at least 20% had five or more. Only 25% of cases with T1D were free from cardiovascular comorbidities, as compared with 35% among cases without T1D.

### Survival analysis

**Figure 2:**
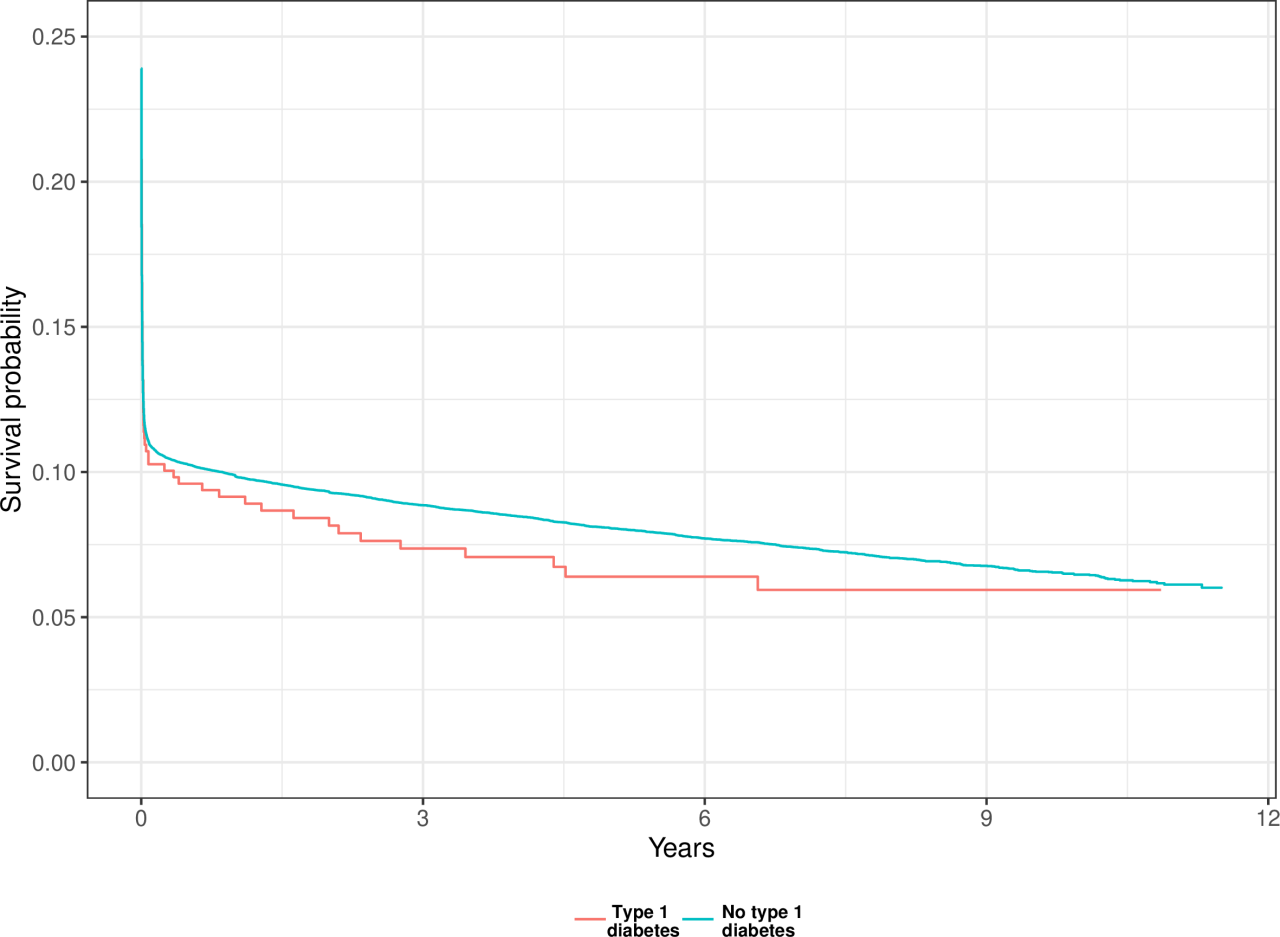
Unadjusted Kaplan-Meier curve.

Figure 2 shows the unadjusted Kaplan-Meier curves for long-term survival. Individuals with T1D showed no significant difference in long-term survival outcome. The log-rank test yielded a p value of 0.83.

Adjusted logistic regression for 30-day survival is presented in table 2. T1D status showed no significant association with survival after accounting for age, initial rhythm, sex and cause of cardiac arrest. Patients with pulseless electrical activity (PEA) and asystole had lower odds of survival compared with ventricular fibrillation/pulseless ventricular tachycardia (VF/pVT).

**Table 2:** Adjusted logistic regression for 30-day survival

Characteristic	OR	95% CI	P value
T1D*	0.7
T1D	–	–
No T1D	0.99	0.96 to 1.02
**Initial rhythm**	<0.001
VF/pVT†	–	–
PEA‡	0.77	0.76 to 0.78
Asystole	0.74	0.74 to 0.75
**Sex**	0.007
Men	–	–
Women	1.01	1.00 to 1.01
**Age**	1.00	1.00 to 1.00	<0.001
**Time from arrest to CPR§ start**	1.00	1.00 to 1.00	<0.001
**Cause of cardiac arrest**	<0.001
Heart disease	–	–
Overdose or intoxication	0.99	0.97 to 1.01
Trauma or accident	0.94	0.92 to 0.96
Pulmonary disease	0.99	0.97 to 1.00
Suffocation	1.00	0.98 to 1.01
Suicide	0.93	0.91 to 0.95
Drowning	1.01	0.98 to 1.04
Other	0.97	0.97 to 0.98

* Type 1 diabetes.

† Ventricular fibrillation/pulseless ventricular tachycardia.

‡ Pulseless electrical activity.

§ Cardiopulmonary resuscitation.

T1Dtype 1 diabetes

## Discussion

A total of 448 cases of OHCA with pre-existing T1D were studied. There was no statistically significant difference in long-term or short-term survival between patients with and without T1D. We found that people with T1D are, on average, 5 years younger when experiencing an OHCA. The cause of arrest was comparatively less attributed to heart disease among cases with T1D (table 1). No significant difference could be seen in the proportion of discharged alive, with patients with T1D at 37.3% compared with 46% among cases without T1D. Both cohort groups exhibited good neurological outcome, with the majority being categorised as either CPC category 1 or 2 (table 3).

**Table 3:** Outcomes following out-of-hospital cardiac arrest

	T1D*	No T1D	P value
Outcomes - n (%)			
Discharged alive	38 (37.3)	5212 (46.0)	0.094
CPC† score at discharge			0.319
CPC score 1 no sequela	26 (76.5)	3346 (75.8)	
CPC score 2 mild sequela	8 (23.5)	666 (15.1)	
CPC score 3 moderate sequela	0 (0.0)	287 (6.5)	
CPC score 4 severe sequela	0 (0.0)	104 (2.4)	
Survival at 30 days	46 (10.3)	5971 (11.0)	0.661

Outcomes are shown in table 3. Patients with T1D that were discharged alive in 37.3% of cases, compared tocompared with 46% of patients without T1D. Patients with T1D had generally good neurological outcomes following OHCAout-of-hospital cardiac arrest, with 76.5% classified as category 1 and 23.5% as category 2. This was also true for patients without T1D. Survival at 30 days was noted for 10.3% of those with T1D compared tocompared with 11.0% of those without T1D.

* Type 1 diabetes.

† Cerebral performance category.

CPCcerebral performance categoryT1Dtype 1 diabetes

As evident in table 1, the prevalence of T1D in this study (0.8%) was higher than that in the general population (0.5%), although proportionally not as high as T2D, which in this study (19%) showed a fourfold higher prevalence in OHCA compared with the Swedish population.^9^ Thus, we do not see an excessive over-representation of T1D in the OHCA population. This should not be taken as evidence of a near-normal risk of OHCA in T1D. A previous study reports the risk of death among individuals with T1D, from any cause and from cardiovascular complications, is twice that of the general population, even with glycaemic control on target.^10^

In line with previous studies, we expected T1D cases to have a higher burden of cardiac aetiologies in OHCA. However, that was not the case in this study. Cardiac aetiologies were less common in T1D. The lower rates of cardiovascular complications likely reflect the advancements in integrated patient care for chronic diseases, improvements in patient education and the management and early treatment of cardiovascular risk factors.^11^ Notably, T1D cases were much more likely to experience an OHCA due to an unspecified (other) cause, compared with non-diabetics. Unfortunately, the SCRR does not provide further details on this specific category, but it is possible that a number of these cases are dead-in-bed syndrome, hypoglycaemic events, hyperglycaemic crisis or other diabetes-related complications. The speculation that dead-in-bed syndrome may explain a larger proportion of OHCA cases in T1D is further supported by the fact that a significantly larger proportion of OHCAs occurred early in the morning hours in people with T1D compared with cases without T1D (table 1).

Moreover, we show that while individuals with T1D are, on average, 5 years younger at OHCA occurrence, they exhibit more comorbidities. Only 25% were free from cardiovascular conditions prior to OHCA compared with 35% among individuals without T1D, including those with T2D (figure 1). This indicates the debilitating nature of T1D at a younger age. Yet, there were no statistically significant associations between T1D and survival. We believe this is due to the relatively small sample size of T1D cases. Although patients with T1D exhibit worse characteristics, p values are overly influenced by sample size and, accordingly, do not favour the survival analysis in this study (figure 2) (table 2).

Additionally, we did not accurately represent the general population by excluding T2D from the control group. The expected variance in survival that would materialise due to this exclusion is marginal and remains statistically insignificant; however, T2D has been previously documented to negatively impact OHCA survival. Its high prevalence among cases without T1D should be taken into consideration when interpreting the disparities.^412^,^14^

The high prevalence of T2D could partly be a consequence of an inherent limitation with register studies and administrative data, predominantly, coding errors and misclassification. The SCRR does not provide us with details regarding the diabetes diagnosis, making us rely on ICD-10 codes. A total of 3380 patients were registered with both E10 (T1D) and E11 (T2D) and consequently were excluded from the T1D group.

This study had no exclusion criteria. Previous reports have had different inclusion criteria for the study population. Some studies included only patients with cardiac arrest of presumed cardiac origin,^13 15 16^ while others included only patients who have survived to hospital admission.^17^ These criteria aimed to create more homogeneous cohort groups, making it easier to isolate the effect of diabetes on survival. However, disregarding all pre-hospital mortality may not only confound the effect of diabetes among survivors but also suggests predictors other than diabetes status have greater importance for prehospital survival. Parry *et al* reported worse outcomes in patients with diabetes following OHCA but noted that these disparities were absent on accounting for initial rhythm.^5^ Consistent with prior investigations, our study found the initial rhythm to be significantly associated with both short-term and long-term survival outcome (table 2).^2 18^ Similarly, our analysis found lower rates of initial shockable rhythm among diabetics, which were not explained by delays in EMS arrival.^5^

A major limitation of this study remains: the SCRR only includes cases in which resuscitation was attempted, excluding all cases in which resuscitation was deemed futile on EMS arrival. It is possible that prehospital mortality not accounted for by the SCRR may include patients suffering OHCA due to complications of diabetes, thus conflicting our ability to draw causal inference.

We conclude that, with the current sample size, there is no statistically significant association between T1D and survival following OHCA. People with T1D are, on average, 5 years younger and have more cardiovascular comorbidities. Patients with T1D present with fewer cardiac aetiologies and shockable rhythm in OHCA. Although predictors such as initial rhythm are more significant than T1D with regards to survival, the risk of SCA remains higher among T1D. Further knowledge may encourage the development of interventions and guidelines to improve prevention and survival in this population.

Future research could potentially elucidate a significant association between T1D and survival following OHCA with a larger sample size. Additionally, comparing the survival rates between patients with T1D and T2D could provide further insights.

## supplementary material

10.1136/bmjopen-2023-080710 online supplemental file 1

## Data Availability

All data relevant to the study are included in the article or uploaded as supplementary information.
