# Supplementary material for: Effects of pre-existing type 1 diabetes mellitus on survival outcome following out-of-hospital cardiac arrest: a registry-based observational study in Sweden
Source: BMJ Open. 2024 Jul 15;14(7):e080710. doi: 10.1136/bmjopen-2023-080710 (PMC11253740; doi:10.1136/bmjopen-2023-080710)
Supplement: online supplemental file 1 [file bmjopen-14-7-s001.pdf]

Supplementary table 1
Baseline characteristics in 54,568 patients with out of hospital cardiac arrest stratified by pre-exististence of type 1 diabetes.

	Type 1 diabetes	No type 1 diabetes	p
**Socioeconomic status - n (%)**			
**Region of birth**			0.370
Sweden	401 (89.7)	46123 (85.6)	
Denmark Finland Norway Iceland	16 (3.6)	2976 (5.5)	
EU	12 (2.7)	1598 (3.0)	
Europe not EU	8 (1.8)	1138 (2.1)	
North America	0 (0.0)	130 (0.2)	
Asia	6 (1.3)	1301 (2.4)	
Africa	2 (0.4)	362 (0.7)	
South America	2 (0.4)	166 (0.3)	
Other	0 (0.0)	75 (0.1)	
Disposable family income - median IQR[1]	2574.50 [1502.75, 3968.25]	2469.00 [1515.00, 3748.00]	0.353
**Work or profession- n (%)**			
Unemployed	274 (69.0)	37564 (77.2)	
**Educational level- n (%)**			0.382
Pre gymnasium 9 years	95 (23.8)	13755 (27.9)	
Pre gymnasium 9 years	53 (13.2)	6220 (12.6)	
Gymnasium 3 years	124 (31.0)	13770 (27.9)	
Gymnasium 3 years	58 (14.5)	6110 (12.4)	
Post gymnasium 3 years	28 (7.0)	3811 (7.7)	
Post gymnasium 3 years or longer	28 (7.0)	4191 (8.5)	
Research education	2 (0.5)	322 (0.7)	
Unknown	12 (3.0)	1169 (2.4)	
Marital status- n(%)			0.005
Not married	121 (30.0)	11225 (22.3)	
Married	162 (40.2)	22946 (45.7)	
Surviving partner	0 (0.0)	3 (0.0)	
Registered partner	0 (0.0)	16 (0.0)	
Divorced	75 (18.6)	8407 (16.7)	
Divorced partner	0 (0.0)	11 (0.0)	
Widow widower	45 (11.2)	7632 (15.2)	
Previous conditions - n (%)			
Hypertension	274 (61.2)	24183 (44.7)	<0.001
Heart failure	101 (22.5)	12316 (22.8)	0.960
Chronic ischemic heart disease	104 (23.2)	11142 (20.6)	0.190
Atrial fibrillation	74 (16.5)	11124 (20.6)	0.041

Type 2 diabetes	0 (0.0)	10423 (19.3)	<0.001
Dyslipidemia	128 (28.6)	8432 (15.6)	<0.001
Angina, including unstable angina	81 (18.1)	8263 (15.3)	0.114
Alcohol dependency	76 (17.0)	7683 (14.2)	0.109
Acute myocardial infarction	64 (14.3)	7280 (13.5)	0.656
Affective disorders	62 (13.8)	5649 (10.4)	0.024
Renal failure	68 (15.2)	5366 (9.9)	<0.001
Thrombotic stroke	33 (7.4)	4739 (8.8)	0.340
Alzheimers dementia	27 (6.0)	4059 (7.5)	0.276
Aortic stenosis	25 (5.6)	3303 (6.1)	0.718
**Medications prescribed - n (%)**			
Anticoagulant or antiplatelet agent ATC[2] B01	166 (37.1)	19636 (36.3)	0.773
Beta blockers	165 (36.8)	18153 (33.5)	0.156
ACE inhibitor or ARB	201 (44.9)	17690 (32.7)	<0.001
Diuretics	144 (32.1)	14607 (27.0)	0.017
Lipid lowering drugs	165 (36.8)	12801 (23.7)	<0.001
Drugs for acid related disorders	111 (24.8)	10822 (20.0)	0.014
Calcium channel blockers	110 (24.6)	8604 (15.9)	<0.001
Other cardiovascular drugs ATC C01	55 (12.3)	6421 (11.9)	0.845
Antihypertensive drugs ATC C02	11 (2.5)	610 (1.1)	0.016
Abbreviations: [1]Interquartile range, [2]Anatomical Therapeutic Chemical Classification System			
