# Supplementary material for: Effects of pre-existing type 1 diabetes mellitus on survival outcome following out-of-hospital cardiac arrest: a registry-based observational study in Sweden
Source: BMJ Open. 2024 Jul 15;14(7):e080710. doi: 10.1136/bmjopen-2023-080710 (PMC11253740; doi:10.1136/bmjopen-2023-080710)
Supplement: Supplementary file 2 [file bmjopen-2023-080710.reviewer_comments.pdf]

PEER REVIEW HISTORY

BMJ Open publishes all reviews undertaken for accepted manuscripts. Reviewers are asked to complete a checklist review form (**http://bmjopen.bmj.com/site/about/resources/checklist.pdf**) and are provided with free text boxes to elaborate on their assessment. These free text comments are reproduced below.

ARTICLE DETAILS

**TITLE (PROVISIONAL)**	Effects of pre-existing type 1 diabetes mellitus on survival outcome following out-of-hospital cardiac arrest: a registry-based observational study in Sweden.
**AUTHORS**	Eken, Berkan; Rawshani, Araz; Rawshani, Aidin; Mandalenakis, Zacharias; Thunstrom, Erik; Louca, Antros; Petursson, Petur; Angerås, Oskar; Nadhir, Sadek; Dworeck, Christian; Råmunddal, Truls

VERSION 1 – REVIEW

**REVIEWER**	Chocron, Richard<br>Paris Centre of Cardiovascular Research
**REVIEW RETURNED**	24-Oct-2023

**GENERAL COMMENTS**	-Can you provide information about the Swedish EMS system?<br>-Can you explain the unemployed rate is very high in the 2 groups (Table 1)?<br>-The noT1D group includes 19.3% of T2D. This proportion is not negliable and you should take into consideration in your main analysis.<br>-You have inlcuded traumatic causes of OHCA! Do you think it is logical to include traumatic CA to explore association between T1D and OHCA? I think you should perform all your analysis with only non traumatic OHCA (by exlcuding traumatic causes)?

**REVIEWER**	Tsuchiya, Asuka<br>University of Tokyo, Department of Clinical Epidemiology and Health Economics
**REVIEW RETURNED**	29-Oct-2023

**GENERAL COMMENTS**	Dear authors<br>Thank you for giving me the opportunity to review this interesting manuscript. This theme is very important.<br><br>Specific comments:<br><br>Methods:<br>1. Page4, line21; maybe a typo, such that ten 10 period<br>2. Please describe the Patient Registry in detail.<br><br>Results:

	3. Talbe1: Please write down all the abbreviations, such as T1D, SMD, ATC, VF, PEA, EMS, and ROSC. Tables and Figures should be self-explanatory.<br>4. Table2 and Table4; Same as table1.<br>5. Table 4: Please change the table title to “Adjusted logistic regression for 30-day survival.” You should make a clear title for readers.<br><br>Discussion;<br>6. “The SCRR only includes casein whom resuscitation was deemed reasonable by the emergency medical service (EMS), excluding all cases in whom resuscitation was deemed futile at the EMS arrival.” This information is VERY important for your cohort. So, move or add this sentence to the Methods section.<br>7. What competing risk do you think? Please describe examples. “Moreover, there may be significant competing risk that is not accounted for in this study.”<br>Please describe the pattern that clearly indicates T1D does confer worse outcomes in OHCA and worse characteristics. “it is reasonable to focus on the patterns in the data instead. The latter indicates rather clearly that T1D does confer worse outcomes in OHCA and worse characteristics.”<br>8. “Disregarding all pre-hospital mortality although may quarantine the effect of diabetes among survivors, also suggests predictors other than diabetes status have greater importance to pre-hospital survival.”<br>9. “Disregarding all pre-hospital mortality” is a considerable limitation of your results because the cohort that EMS decided not to attempt CPR may contain more T1D patients.<br>10. “T1D are less likely to survive following OHCA.” This conclusion does not stand for your result.

**REVIEWER**	Lin, Steve<br>University of Toronto
**REVIEW RETURNED**	05-Nov-2023

**GENERAL COMMENTS**	Thank for the opportunity to review this observational study describing the characteristics of T1DM patients with OHCA. The study provides some interesting results and is additive to the body of literature. However, there are a number of general comments and suggestions that I have for the authors to consider.<br><br>1) Although it is important to separate T1DM from T2DM as it relates to OHCA, the authors rightfully point out that the sample size is small using their methods of identification and inclusion. I personally believe that the study would be more robust to include all DM patients and include the separation of T1DM and T2DM as secondary analyses.<br><br>2) Using administrative data to identify T1DM has its limitations since there are inherent risks of coding errors, particularly when only including E10. T1DM patients could also be coded as E12-E14 or not at all. This should be further discussed in the manuscript.

	3) Although T1DM patients have an increased risk of CVD despite treatment, improved glucose control may also help explain the results of lower heart disease in T1DM patients compared to no T1DM. This should be further discussed.<br><br>4) Table 1 is very large. Suggest splitting into two tables.<br><br>5) The conclusions do not necessarily represent the results. Survival at discharge is not significantly lower and survival at 30 days is very similar. This may be related to the identification of the study population.

VERSION 1 – AUTHOR RESPONSE

Dr. Richard Chocron
Dear colleague,
Thank you for reviewing our manuscript.

- Further information regarding the Swedish EMS system is now provided in the Methods section.
- The unemployment rate could not be further explained with the available data.
- We agree that proportion of T2D is not negligible, it is now mentioned in the main analysis.
- It is discussed in the main analyses that excluding cases could isolate the effect of T1D however we feel, given the small number of traumatic cases, the results would not change.

Dr. Asuka
Dear colleague,
Thank you for reviewing our manuscript so thoroughly. It is much appreciated.

- Specific typos are now corrected.
- Tables and figures are now self-explanatory, abbreviations are included in all.
- The patient registry is further described.
- "The SCRR only includes casein whom resuscitation was deemed reasonable by the emergency medical service (EMS), excluding all cases in whom resuscitation was deemed futile at the EMS arrival." Is now included in the Methods section and is further discussed in the Discussion section.
- Sentence regarding competing risk has been removed.
- We have added to Discussion regarding the cohort, rejected by EMS, also may contain T1D patients.
- The conclusion has been adjusted to better represent the results.

Dr. Steve Lin
Dear colleague,
Thank you for your review of our manuscript. Your suggestions have been taken into consideration.

- Although your suggestion to include all DM patients in our analyses is reasonable our purpose for this manuscript is to fully focus on T1D.

- The limitation of administrative data and coding errors is now mentioned in the Discussion section.
- Added to Discussion with regards to explanation of lower cardiac etiologies, we agree with your comment.
- Table 1 has been reduced and the redacted part is presented as a supplementary table.
- The conclusion has been adjusted to better represent the results.

VERSION 2 – REVIEW

**REVIEWER**	Chocron, Richard<br>Paris Centre of Cardiovascular Research
**REVIEW RETURNED**	18-Mar-2024

**GENERAL COMMENTS**	The authors addressed my comments. The article is now acceptable for publication

**REVIEWER**	Tsuchiya, Asuka<br>University of Tokyo, Department of Clinical Epidemiology and Health Economics
**REVIEW RETURNED**	18-Mar-2024

**GENERAL COMMENTS**	Well revised.<br>I have no comment any more.

VERSION 2 – AUTHOR RESPONSE
